# Experimental colitis delays and reduces the severity of collagen-induced arthritis in mice

**DOI:** 10.1371/journal.pone.0184624

**Published:** 2017-09-19

**Authors:** Julie Hablot, Laurent Peyrin-Biroulet, Tunay Kokten, Reine El Omar, Patrick Netter, Claire Bastien, Jean-Yves Jouzeau, Harry Sokol, David Moulin

**Affiliations:** 1 IMoPA, UMR7365 CNRS-Université de Lorraine, Vandœuvre Les Nancy, France; 2 NGERE, UMR_S954 INSERM-Université de Lorraine, Vandœuvre Les Nancy, France; 3 Service d'hépato-gastroentérologie, CHRU de Nancy, Vandœuvre Les Nancy, France; 4 Service d’anatomopathologie, CHRU de Nancy, Vandœuvre Les Nancy, France; 5 Department of Gastroenterology, Saint Antoine Hospital, Paris, France; 6 Sorbonne University—UPMC Université Paris 06, INSERM ERL 1157, Avenir Team Gut Microbiota and Immunity, UMR 7203 CNRS, Saint-Antoine Hospital, AP-HP, Paris, France; 7 Micalis Institute, UMR INRA—AgroParisTech, Université Paris-Saclay, Jouy-en-Josas, France; 8 CHRU de Nancy, Contrat d’interface, Vandœuvre Les Nancy, France; "INSERM", FRANCE

## Abstract

Amongst extraintestinal manifestations (EIM) occurring in IBD patients, rheumatologic manifestations are the most frequent. Understanding the relationships between arthritis and colitis is a prerequisite to improving the management of these patients. Microbiota of patients with IBD or rheumatologic diseases, like spondyloarthritis (SpA) is modified compared to healthy individual. Thus, we have evaluated the impact of colitis in the development of arthritis in mice and we have analyzed microbiota changes. Collagen-induced arthritis (CIA) was induced at day 0 in DBA1 mice exposed or not to Dextran Sodium Sulfate (DSS) to induce colitis between day 14 and day 21. Animals were monitored regularly for arthritis and colitis severity (clinical score, hindpaw edema). Fecal microbiota was studied by 16S rRNA deep sequencing at critical time points (D14, D14, D21 & D41). At day 41, histological scoring of the intestines and ankles were performed at the end of experiment. Induction of colitis slightly delayed arthritis onset (2 ± 1 days of delay) and reduced its severity (5.75 ± 1.62 in arthritis only group vs 4.00 ± 1.48 in arthritis + colitis group (*p* = 0.02 at day 28) macroscopically and histologically. In contrast, colitis severity was not influenced by arthritis development. Induction of colitis promoted a modification of microbiota composition and a decrease of α-diversity. Fecal microbiota composition was different between “colitis” and “arthritis+colitis” groups during colitis development. Interestingly a milder decrease of bacterial diversity in the “arthritis+colitis” group was observed. Concomitant experimental colitis protects mice against collagen-induced arthritis and this is associated with changes in gut microbiome composition.

## Introduction

Inflammatory bowel diseases (IBD) affecting over 1 million individuals in the USA and 2.5 million in Europe [1]. The two main IBD are Crohn’s disease (CD) and ulcerative colitis (UC). IBD are characterized by a chronic inflammation of intestinal mucosa. Patients with IBD, known to be systemic disorders, are likely to develop extraintestinal manifestations (EIMs). EIMs have a prevalence rate ranging from 6% to 47%. Approximately one third of IBD patients will develop EIMs in the course of their disease [2–4]. In a cohort study of 950 IBD patients, 43% of CD patients and 31% of UC patients had EIMs [5]. The prevalence of EIMs is higher in CD compared to UC [5,6]. The development of one EIM appears to increase the risk of developing another EIM [7]. The treatment of EIM in IBD patients remains challenging in clinical practice and often requires the use of biologics raising both safety and cost issues. Joint involvement (Spondyloarthritis SpA) is the most common EIM in patients with IBD, with a prevalence ranging between 17% and 39% [8]. This comorbidity can be very disabling and is associated with a more severe disease course in IBD patients [5].

Two main types of rheumatism can be associated with IBD: *peripheral arthritis* such as synovitis and/or dactylitis and/or enthesopathy and *axial involvement* [9]. Two subtypes categorize the peripheral arthritis: type 1, the pauci-articular form, generally running parallel to the intestinal disease, is acute and self-limiting; and type 2, the polyarticular form, running independently from IBD, with symptoms lasting for months or years [10].

Evidence from experimental and clinical studies have implicated gut microbiota in both IBD and SpA pathophysiology[11]. Decrease of the diversity of bacteria, has been observed in experimental and clinical situations for IBD [12] and SpA [13]. This dysbiosis is thought to promote an imbalance between pro-inflammatory and anti-inflammatory cells in gastrointestinal tract. Induction of colitis with dextran sodium sulfate (DSS) in mice, promotes changes in the gut microbiota diversity [14], and involves immune system [15]. HLA-B27 transgenic rats, a SpA model, have a different microbiota compared to the non-transgenic rats [16,17]. These transgenic rats spontaneously not only develop a SpA but also a colitis representing human disease. In collagen-induced arthritis (CIA) model in mice, a disturbance of gut microbiota modifies the severity of disease [18].

The reciprocal influence of colitis and arthritis is unknown. We evaluated for the first time, the impact of experimental colitis in the development of collagen-induced arthritis in mice and the associated gut microbiota changes.

## Materials and methods

### Induction of colitis and arthritis

**Fig 1:**
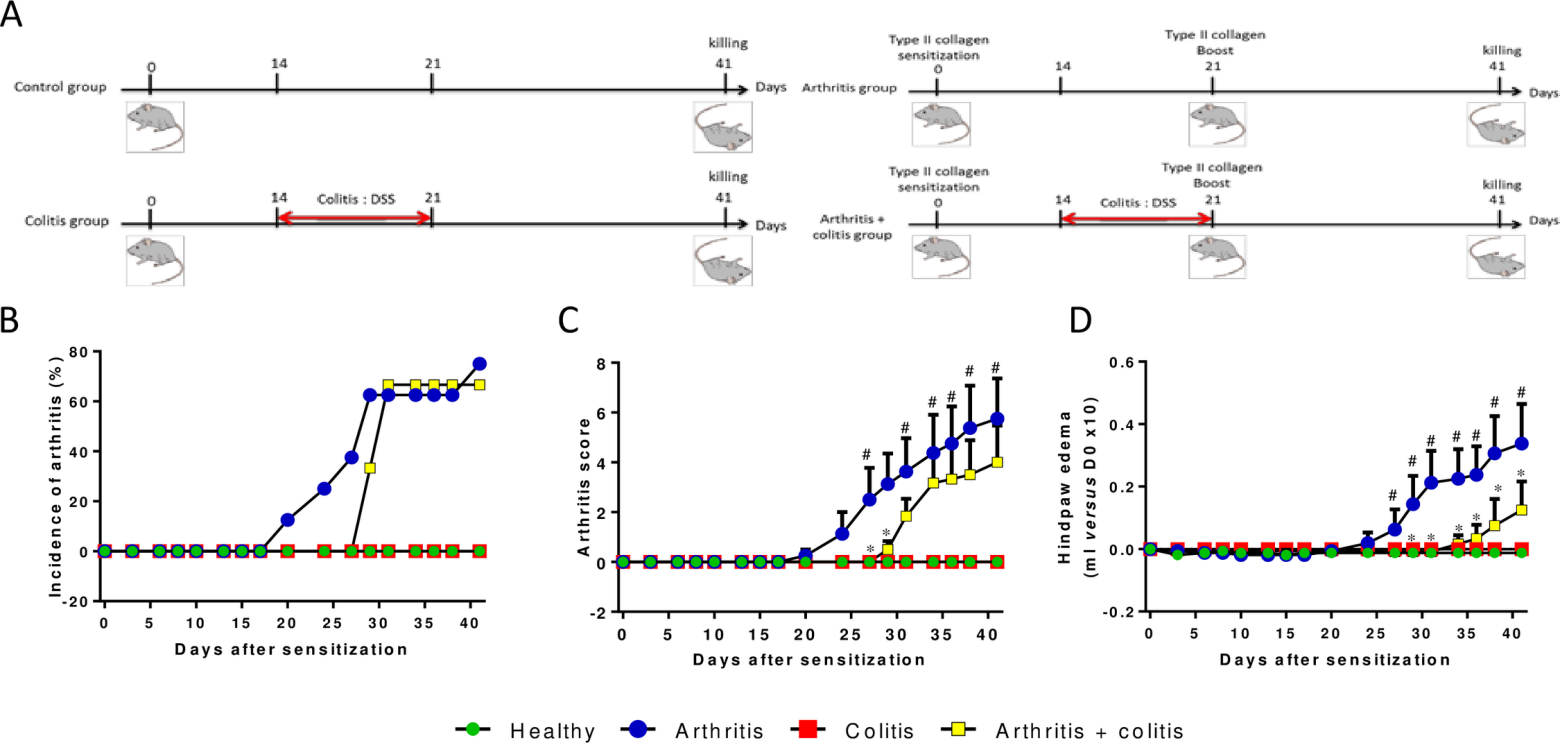
Colitis occurrence reduces arthritis severity. (A) Schematic representation of study design. Incidence of arthritis has been monitored (B) and clinical parameters, arthritis score (C) and hindpaws volume (D), have been measured in control, arthritic, colitic or arthritic and colitic mice (N = 8 by group). Arthritis was induced by injection of CII (200μg) at D0 at the basis of the tail and a boost of 100μg CII was performed at D21 by intra-peritoneal injection. Colitis was induced by oral intake of 3% DSS in drinking water from D14 to D21. Data are expressed as mean ± SEM. # P<0.05 arthritis group *versus* control group. * P<0.05 arthritis + colitis group *versus* arthritis group.

The protocol was developed according to local and international recommendations and all experiments were monitored by the staff of the animal facility, which was authorized (agreement # 154-547-025) local ethics committee (CEMLEA Comité d'éthique en Matière d'Expérimentation Animale). Surgery and necropsy were performed under ketamine/acepromazine anesthesia, and all efforts were made to minimize suffering. Collagen-induced arthritis was induced in 7-weeks old DBA/1 male mice (Janvier Labs). At day 0, animals were sensitized at the basis of the tail by intradermal injection of 100μl of type II bovine collagen (CII, MDbioproducts) suspension at 2mg/ml and emulsified in complete Freund’s adjuvant (CFA). At day 21, a booster ip injection of 100μl collagen II mixture at 1mg/ml, was performed. Colitis was induced by administration of 3% DSS (Colitis grade MW 36-50kDa) (MPBiomedicals) in drinking water for 7 days. Animals were housed in groups of five in solid-bottomed plastic cages with access to tap water and standard rodent pelleted chow (Scientific Animal Food & Engineering A04) *ad libitum*. Three experimental groups were studied: CIA alone (referred as arthritis), DSS colitis alone (referred as colitis), and CIA in animals exposed to DSS during arthritis development (referred as arthritis + colitis). One group of DBA/1 mice was used as control. Study design is summarized on Fig 1A.

### Clinical assessment of arthritis

Mice were weighed daily. The severity of arthritis was evaluated every day on each paw with a score from 0 to 4, for a maximal score of 16 by animal (0 = normal joint, 1 = moderate redness and slight swelling or swelling of one or two digits, 2 = moderate redness and moderate paw swelling or swelling more than three digits, 3 = redness and swelling from tarsal joints to metatarsal joints, 4 = severe redness and severe swelling of the entire paw).

Hindpaw swelling was measured at the same time by plethysmography. Paw volume determined prior to disease induction was used as the baseline.

### Histological assessment of arthritis

The development of arthritis was analyzed by histological assessment. Mice were killed by cervical dislocation and hindpaws were dissected then fixed in 4% formalin (Labonord). Hindpaws were further decalcified in 10% EDTA for 1 month and embedded in paraffin blocks. Five μm-thick tissue sections were cut and two strainings were performed, hematoxylin, eosin and safran and toluidine blue. Histological examination was carried out blindly by three independent observers. Synovium from ankle joint was graded on a scale of 0 to 3 (with 0 = normal and 3 = major changes) for synoviocyte hyperplasia (depth of lining layer) and tissue cell infiltration. Cartilage degradation was graded from 0 = fully stained cartilage, 1 = destained cartilage, 2 = destained cartilage with synovial cells invasion to 3 = complete loss of cartilage. The following morphologic criteria have been used for bone erosion: 0 = normal, 1 = mild loss of cortical bone at few sites, 2 = moderate loss of cortical and trabecular bone and 3 = marked loss of bone at many sites.

For each group, 8 sections were graded at different fields to provide representative samples of the whole joint. Mean scores have been determined from the different sections of the individual animals allowing the calculation of composite scores for the different experimental groups.

### Measurement of mediator expression by reverse transcription polymerase chain reaction (RT-PCR)

At necropsy, forepaws of mice were preserved in trizol™ then were frozen at -80°C until use. Forepaws were grinded with homogenizer-disperser tool (Ultra-turrax, IKA) in 3ml of trizol. Samples were centrifuged at 12,000g for 10min at 4°C and 200μl of chloroform per milliliter of trizol™ were added to the supernatant. Samples were centrifuged at 12000g for 15min at 4°C, 500μl of isopropanol per milliliter of trizol™ were added to the aqueous phase before incubation overnight at -20°C. RNAs were pelleted by centrifugation at 12000g for 10min and were washed twice with 70% ethanol. Finally, RNAs were taken up in water.

Reverse transcription was performed with 2μg of RNA, 4μl of RT buffer, 2μl of dNTP at 5mM, 2μl of DTT at 100mM, 1μl of hexa primers at 0,2μg/μl and 1μl of M-MLV at 200u/μl (Invitrogen). Samples were incubated at 37°C for 1h30 and at 95°C for 5min.

For PCR, samples were diluted to one-tenth for S29, chosen as a housekeeping gene, and one-fifth for genes of interest (TNFα, IL-1β, iNOS), respectively. PCR was performed with 5μl of samples, 10μl of SYBR, 1μl of each primer at 10μM and 3μl of water. Sequences of primers are listed in S1 Table.

### Gut microbiota analysis

Feces were freshly harvested then frozen at -80°C until use.

#### Fecal DNA extraction

Genomic DNA was extracted from 200 mg of feces as previously described [19]. Following microbial lysis involving both mechanical and chemical steps, nucleic acids were precipitated via isopropanol for 10 minutes at room temperature, followed by incubation for 15 minutes on ice and centrifugation for 30 minutes at 15,000 *g* and 4°C. Pellets were suspended in 112 μL of phosphate buffer and 12 μL of potassium acetate. After the RNase treatment and DNA precipitation, nucleic acids were recovered via centrifugation at 15,000 *g* and 4°C for 30 minutes. The DNA pellet was suspended in 100 μL of TE buffer.

#### 16S rRNA gene sequencing

A 16S rRNA gene fragment comprising the V3 and V4 hypervariable regions (16S (sense) 5’-TACGGRAGGCAGCAG-3’ and (antisense) 5’-CTACCNGGGTATCTAAT-3’) was amplified using an optimized and standardized 16S-amplicon-library preparation protocol (Metabiote, GenoScreen, Lille, France) as recently described [20]. Briefly, 16S rRNA gene PCR was performed using 5 ng genomic DNA according to the manufacturer’s protocol (Metabiote) using 192 bar-coded primers (Metabiote MiSeq Primers) at final concentrations of 0.2 μM and an annealing temperature of 50°C for 30 cycles. The PCR products were purified using an Agencourt AMPure XP-PCR Purification system (Beckman Coulter, Brea, CA, USA), quantified according to the manufacturer’s protocol, and multiplexed at equal concentrations. Sequencing was performed using a 300-bp paired-end sequencing protocol on an Illumina MiSeq platform (Illumina, San Diego, CA, USA) at GenoScreen, Lille, France as described previously [20].

#### Processing of sequences

The sequences were demultiplexed, quality-filtered using the ‘quantitative insights into microbial ecology’ (QIIME, version 1.9.1) software package, and the forward and reverse Illumina reads were joined using the fastq-join method (http://code.google/p/ea-utils). The sequences were assigned to OTUs using the UCLUST algorithm [21] with a 97% threshold of pairwise identity and classified taxonomically using the Greengenes reference database [22]. Rarefaction was performed (25,000 sequences per sample) and used to compare the abundances of OTUs across samples.

Principal component analyses (PCA) of the Unweighted Unifrac distance with each sample colored according to the disease phenotype were built and used to assess the variation between experimental groups. The number of observed species, as well as the Shannon, Simpson and Chao1 diversity indexes were calculated using rarefied data (depth = 25,000 sequences/sample) and used to characterize species diversity in a community. Differential analysis was performed using the linear discriminant analysis (LDA) effect size (LEfSe) pipeline [23].

### Statistics

Data are expressed as mean ± SEM. Arthritis score and histological grading were analysed with the Kruskall-Wallis test, using StatView™ version 5.0 software (SAS Institute Inc., Cary, NC, USA). All other data were compared by analysis of variance (ANOVA) followed by Bonferroni *post-hoc* test. Differences were considered significant at *P* < 0.05.

## Results

### Colitis dampened collagen-induced arthritis severity

No sign of arthritis was observed in control and “colitis” groups. In the “arthritis” group, the first arthritis symptoms were observed by day 20 whereas arthritis developed 8 days later in the “arthritis + colitis” group. (Fig 1B). Arthritis severity was moderate with a maximum mean clinical score of 5.8 ± 1.6 in the “arthritis” group *vs* 4.0 ± 1.5 in the “arthritis + colitis” group (Fig 1C). Hindpaw edema increased gradually from day 24 to day 41 in the “arthritis” group, whereas oedema occurred by day 34 in the “arthritis + colitis” group and remained significantly less severe (Fig 1D).

**Fig 2:**
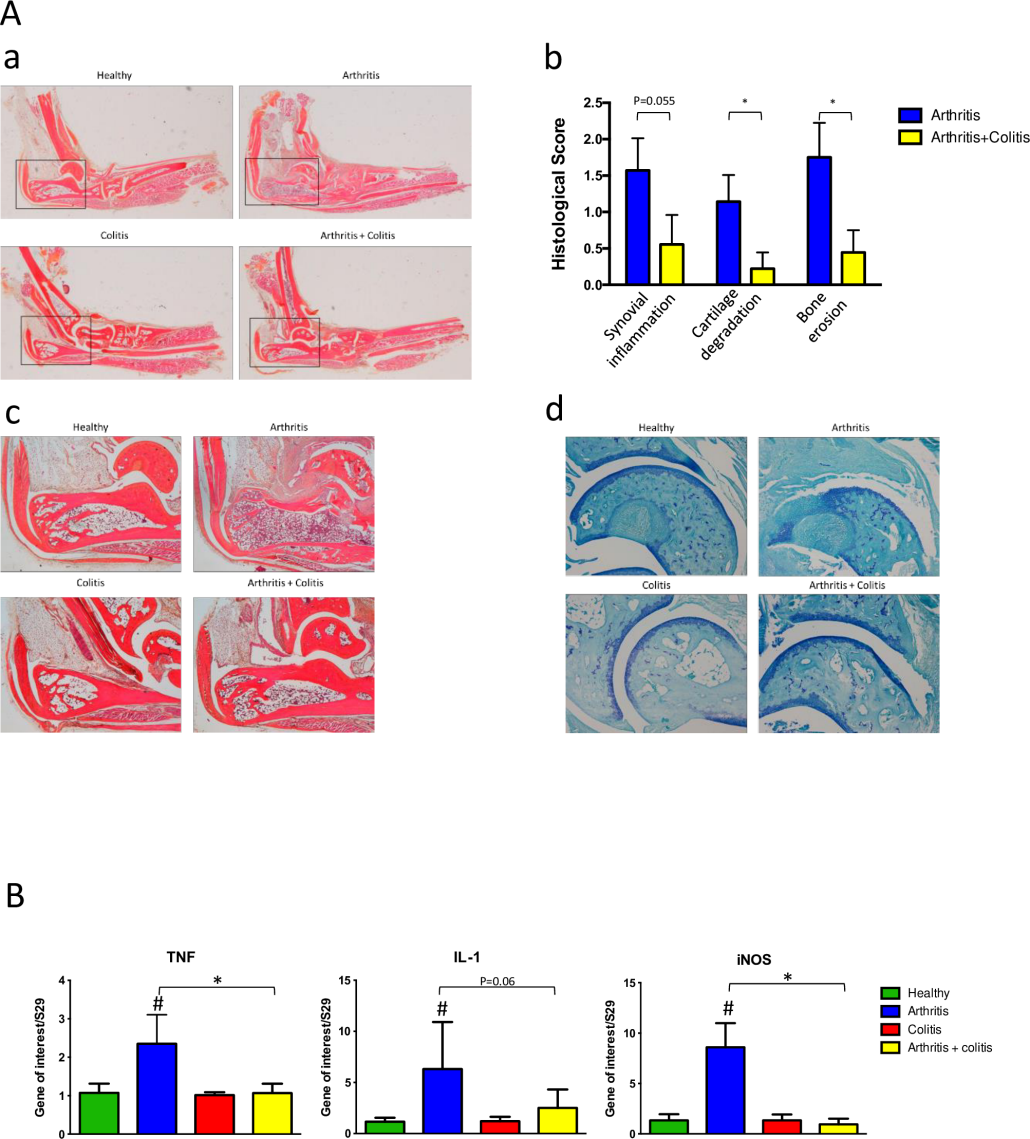
Analysis of joint inflammation and structural articular degradation in mice. Analyses were performed 41 days after arthritis sensitization and/or 27 days after colitis induction (N = 8 by group). (Aa) Macroscopic examination of ankle inflammation (Ab), histological grading of synovium infiltration and hyperplasia, cartilage degradation and bone erosion, (Ac) representative picture of ankles section (HES staining, X4 magnification) (Ad) representative sections of articular cartilage (toluidine blue staining, X10 magnification). (B) Measurement inflammatory cytokines and inflammation marker in forepaws (n = 5 by group) on day 41 by real time PCR. Data are expressed as mean ± SEM, # P<0.05 arthritis group *versus* control group, * P<0.05 arthritis + colitis group *versus* arthritis group.

These observations were confirmed by joint histological analysis at necropsy (Fig 2A). As expected, ankles of mice showed no sign of inflammation or cartilage degradation in control and colitis groups, whereas 80% of ankles of mice in “arthritis” group *vs* 40% in “arthritis+ colitis group” were arthritic. Inflammation (synovial hyperplasia and cell infiltration) (Fig 2Ac), and joint structural damages (cartilage degradation (Fig 2Ad) and bone infiltration), were more severe in the arthritis group compared to “arthritis + colitis” group.

The expression of the proinflammatory cytokines TNFα and IL-1β and iNOS gene (chosen as a marker of chronic inflammation) in the forepaws showed an increase in the “arthritis” group whereas no significant changes were observed in the “arthritis + colitis” group.

### Colitis severity was not affected by arthritis

In “control” and “arthritis” groups, no clinical sign of colitis was detected (S1 Fig). In “colitis” and in “arthritis + colitis” groups, mice developed colitis between day 15 and day 27 with a peak at day 20. No statistical significant difference was observed between these two groups (S1A and S1B Fig).

Fecal lipocalin 2, a sensitive and broadly dynamic non-invasive biomarker for intestinal inflammation was measured at the indicated time points. Basal levels of fecal lipocalin-2 were observed in “control” and “arthritis” groups. As expected colitis caused an increase of fecal lipocalin-2 in “colitis” and in “arthritis + colitis” groups (S3 Fig). Colon length was reduced in DSS induced group (S1C Fig). At day 41, the expression of inflammatory genes in colon were similar in all groups and no difference was observed between “colitis” group and “arthritis + colitis” group. (S2 Fig). Colitis was observed histologically in both “colitis” and “arthritis + colitis” group without statistical differences in severity (S1E and S1F Fig).

### Changes in gut microbiota

**Fig 3:**
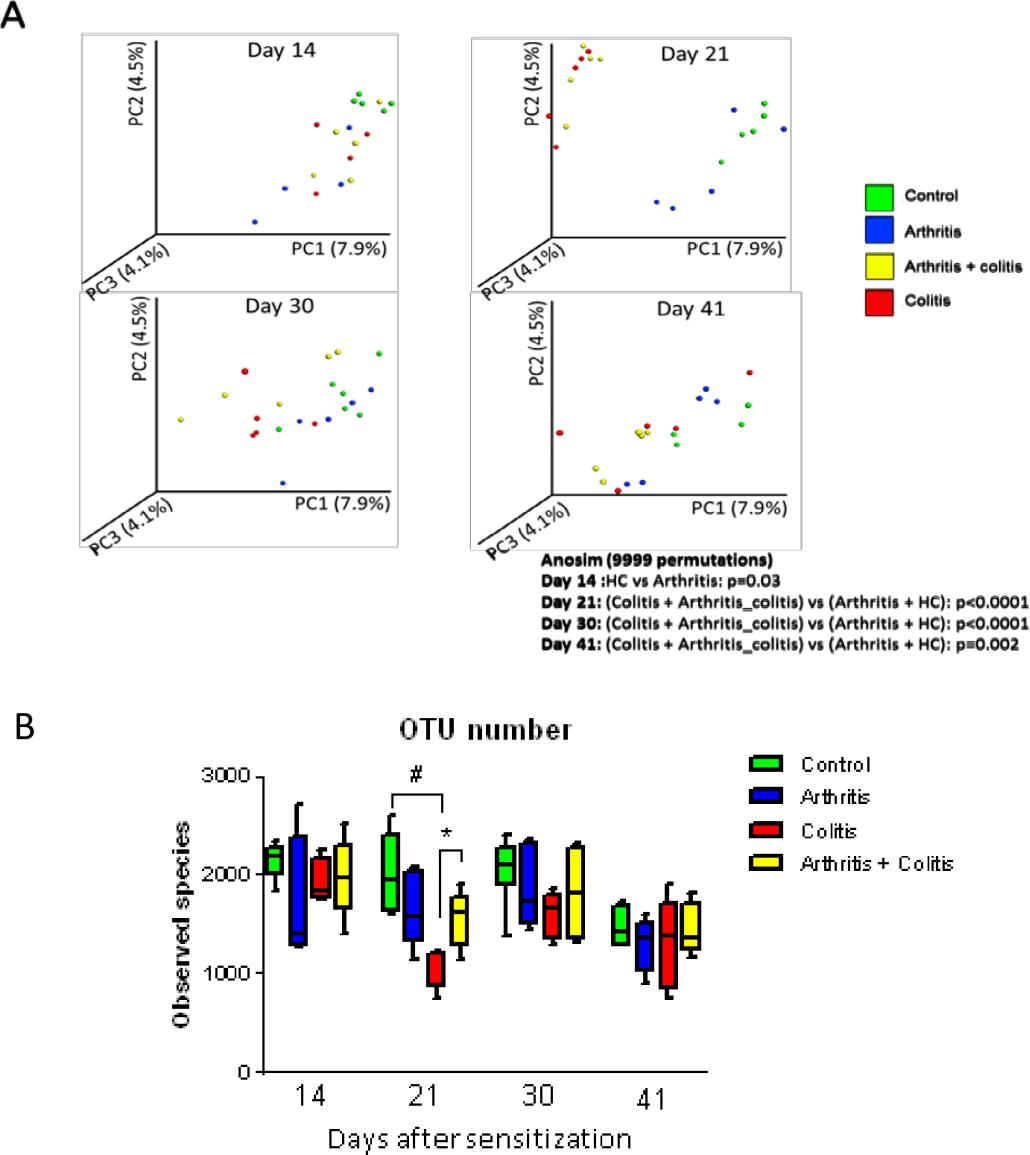
Diversity of fecal microbiota of control, arthritic, colitic or arthritic and colitic mice (N = 5) at days 14 (before induction of colitis), 21 (during colitis), 30 (after colitis) and 41 (after colitis). Arthritis was induced by injection of CII (200μg) at D0 at the basis of the tail and a boost of 100μg CII was performed at D21 by intraperitoneal injection. Colitis was induced by oral intake of 3% DSS in drinking water from D14 to D 21. (A) Representation of β-diversity of fecal microbiota. (B) Representation of α-diversity. Data are expressed as mean ± SEM. # P<0.05 colitis group *versus* control group. * P<0.05 arthritis + colitis group *versus* colitis group.

**Fig 4:**
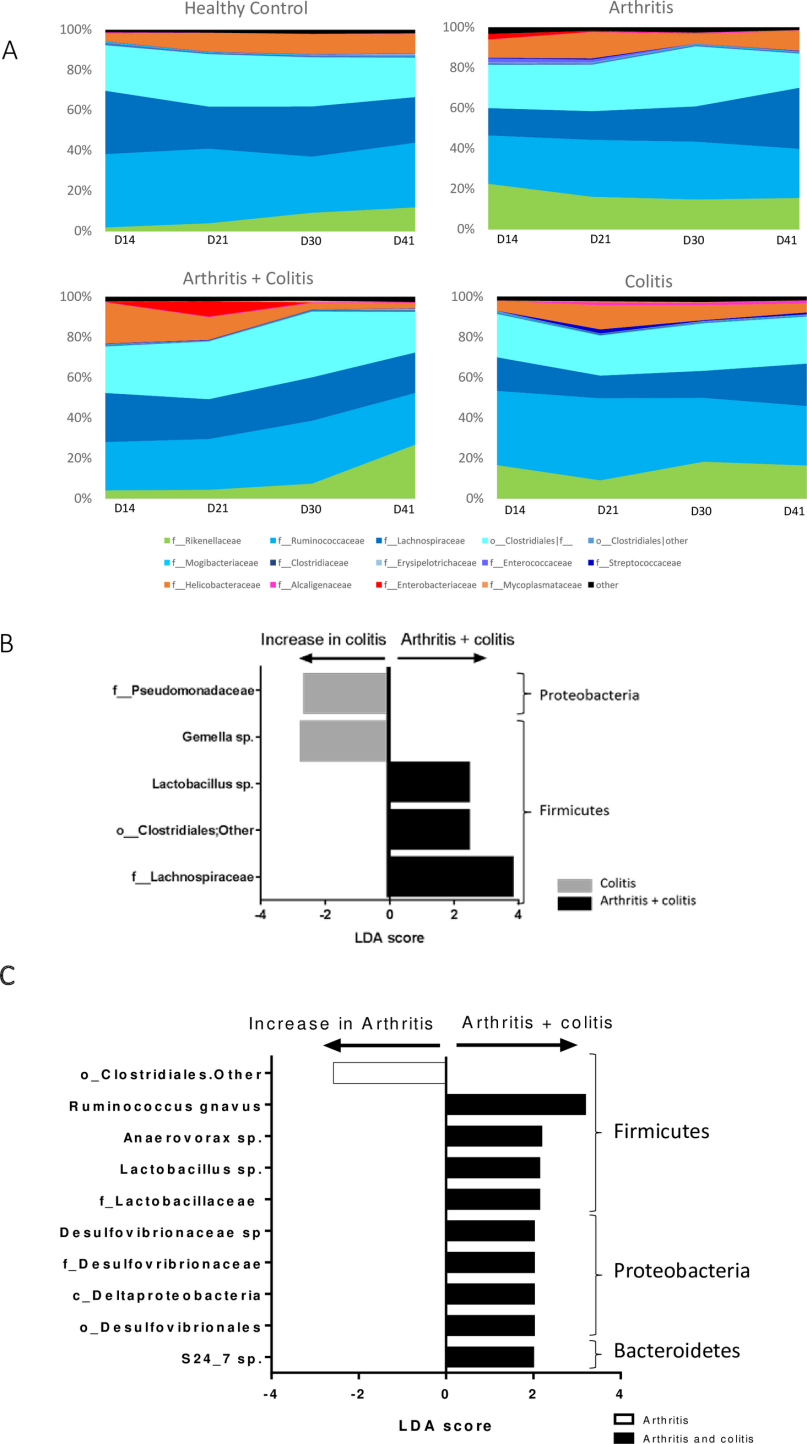
Composition of fecal microbiota in this model. (A) Composition of fecal microbiota (Family level) of control, arthritic, colitic or arthritic and colitic mice (N = 5) at days 14 (before induction of colitis), 21 (during colitis), 30 (after colitis) and 41 (after colitis). (B) Differential analysis between colitis and colitis + arthritis groups at day 21. (C) Differential analysis between arthritis and colitis + arthritis groups at day 21.

Induction of colitis caused changes in fecal microbiota composition. Principal components analysis of beta diversity showed significant differences between “colitis” and “arthritis + colitis” groups compared with “control” and “arthritis” groups at days 21, 30 and 41 (Fig 3A). Induction of arthritis did not modify bacterial α-diversity. This parameter was significantly lower in”colitis” group compared with “arthritis + colitis” group at day 21 (Fig 3B). Fecal microbiota composition was different between “colitis” and “arthritis + colitis” groups, and notably at day 21 (Fig 4A and 4B). At this time point, several taxa belonging to Firmicutes phylum were more present in “arthritis + colitis” group compared to “colitis” group. This was the case for Lactobacillus genera, and Lachnospiraceae family. Conversely, Gemella genera in the Firmicutes phylum and Pseudomonadaceae family in the Proteobacteria phylum, were less represented in “arthritis + colitis” group compared to “colitis” group.

At day 21 and 30, fecal microbiota composition was different between “arthritis” and “arthritis + colitis” groups (Table 1 and Fig 4C). Several taxa belonging to Firmicutes phylum were decreased in “arthritis” group compared to “arthritis + colitis” group such as *Ruminococcus gnavus* and Desulfovibrionaceae family, belonging to Proteobacteria phylum.

**Table 1:** Comparison of β-diversity between “arthritis + colitis” versus “arthritis” alone group. Anosim 9,999 permutations.

	D14	D21	D30	D41
**p value**	0.177	0.0085	0.015	0.064

At day 30 and 41, α-diversity was similar between “arthritis + colitis” group and “arthritis” group and β-diversity did not show any significant difference (Fig 3A and 3B). However, at these time points, few differences were observed in fecal microbiota composition between these two groups (S3 Fig). At day 30, only *Ruminoccocus* genera (Firmicutes phylum), and particularly *R*. *gnavus* species, was more present in “arthritis + colitis” group compared to “arthritis” group. At day 41, in addition to *Ruminoccocus*, *Blautia* and Gemellaceae (belonging to Firmicutes phylum) were more represented in “arthritis + colitis” group compared to “arthritis” group.

## Discussion

IBD is a systemic condition and can be accompanied by extraintestinal manifestations (EIMs) [24]. Articular manifestations are the most frequent EIM; between 17% and 39% of patients with IBD have a spondyloarthritis (SpA) [25]. Understanding the mechanisms underlying the development of such complication in IBD is a prerequisite to improving their management and remains an unmet need for these patients. Herein we show that experimental colitis could affect arthritis development in mice. Indeed, induction of colitis delayed arthritis onset and reduced its severity. We observed a lower arthritis score and edema in arthritis + colitis group compared to arthritis group. In contrast, colitis severity was not strongly influenced by arthritis development. This is, to our best knowledge, the first example of a transorgan effect in experimental IBD.

In healthy adult, gut microbiota is relatively stable and plays a key role in homeostasis of the organism [26,27]. Human and mouse gut microbiota appear to be composed mainly of bacteria from the Firmicutes and Bacteroidetes phyla [28–30]. Several studies showed that gut microbiota is involved in inflammatory and metabolic diseases [26,27] among which IBD and rheumatologic diseases.

Many groups worldwide have pointed out the IBD-associated dysbiosis, which is characterized by a lower biodiversity, a decreased level of some Firmicutes such as *Faecalibacterium prausnitzii* [31] and an increased level of Proteobacteria such as *Escherichia coli* [32]. This imbalance in microbiota composition might have functional consequences as some decreased bacteria such as *F*. *prausnitzii* have been shown to exhibit anti-inflammatory effects [33], whereas other bacteria such as *E*. *coli* have pro-inflammatory effects [34]. As previously observed, fecal microbiota of mice with DSS-induced colitis was characterized by a decreased diversity and changes in microbiota composition. As expected, DSS-induced colitis provoked an increase in the Bacteroidetes over Firmicutes ratio whereas no changes in Proteobacteria phylum was observed [29].

Gut microbiota has several effects on immune cells both within and beyond the gut and could thus play a role in the development of extraintestinal manifestations. There are many recent arguments supporting the role of gut microbiota in the pathogenesis of rheumatic disease (reviewed in[35]).

Majority of microbiota and studies in rheumatic diseases have focused on rheumatoid arthritis, the microbiota of SpA remains largely unknown. Stebbings *et al*. determined the faecal microbiota composition of 15 SpA patients and 15 matched controls using molecular method allowing the identification of few specific bacterial groups[36]. They demonstrated a significantly higher proportion of sulphatereducing bacteria in faecal samples from ankylosing spondylitis (AS) patients compared with samples from controls. More recently, Costello *et al*. showed evidence for a discrete microbial signature in the terminal ileum of patients with axial form of SpA compared with healthy control subjects using the 16rRNA gene sequencing method[37]. The microbial composition was demonstrated to correlate with disease status. Importantly, of the 7 families of microbes with differences in abundance within the SpA patients’ microbiome, *Lachnospiraceae*, *Ruminococcaceae*, and *Prevotellaceae* were strongly associated with colitis and CD, (n = 9 patients and 9 healthy matched controls). However, larger studies are required to define the individual bacterial species involved.

Collagen-induced arthritis (CIA) shares many similarities with human rheumatoid arthritis. Indeed the two major characteristics of the CIA model—breach of tolerance and generation of auto—antibodies toward self and collagen- make CIA the gold standard in vivo model for RA studies, but not for spondyloarthritis. IBD associated arthritis is considered by rheumatologist as spondyloarthritis. The lack of reliable inducible model of SpA in mice[38], or the fact that articular inflammation is concomitantly associated with intestinal inflammation in spontaneous SpA models lead us to choose the CIA model to decipher the impact of digestive inflammation on joint disease and vice versa.

Gut microbiota play an important role in development of inflammatory arthritis in human and in animal models [39], in particular in CIA. During arthritis development, fecal microbiota was different between CIA-susceptible mice and CIA-resistant [40]. Partial depletion of microbiota with antibiotic has been shown to modify the severity of arthritis in this model [18]. Herein, we observed that microbiota composition of mice with arthritis and colitis is different from mice with colitis only. Firmicutes phylum is mainly affects; Lactobacillus genus and Clostridiales order are more present in mice with arthritis and colitis compared to mice with colitis alone. Several studies showed that species from Lactobacillus are beneficial in DSS-induced colitis [41,42]. Thereby, Lactobacillus sp increase in arthritis + colitis group might play a role in the subclinical improvement as observed by the decrease in fecal lipocalin-2 level.

Composition of fecal microbiota was also different between arthritis and arthritis + colitis groups. At arthritis and colitis onset, we observed that Lactobacillaceae, and notably Lactobacillus, *Ruminococcus gnavus* belonging to Lachnospiraceae and S24_7 species belonging to Bacteroidales were more present in mice with arthritis and colitis compared to arthritis group. Interestingly, these groups of bacteria had been shown to be more present in mice with higher susceptibility to arthritis development [40]. Thus, we might hypothesize that colitis has altered the gut microbiota of these mice and thus delayed the appearance of “pro-arthritogenic” bacteria. In a very recent report, Breban and collaborators revealed specific dysbiosis in SpA patient distinctive from dysbiosis found in RA patients and evidence an increase in *Ruminococcus gnavus* that appears specific for SpA and a marker of disease activity, further highlighting the clinical relevance of our findings.

Taken together our results demonstrate that concomitant experimental colitis protects mice against collagen-induced arthritis and this is associated with changes in gut microbiome composition. Some limitations of this study should be considered. Other mechanisms than microbiota changes may contribute to arthritis protection by colitis in this model. We cannot exclude that DSS-induced colitis could induce a “titration” of the immune system (in terms of immune cell mobilization and/or polarization) towards gut inflammation preventing articular disease occurrence. Our data on microbiota are preliminary and would require fecal transplantation experiments to establish a causal link. In this regard, it would be interesting to investigate the distribution of mucosal and systemic Th17 in this model. Indeed IL-17 contributes to both arthritis and colitis in experimental model and in human[43] In physiological conditions, Th17 cells are involved in extracellular pathogens defence and act more specifically in the mucosal firewall[44]. Indeed, in basal conditions most Th17 cells are found in the gastro-intestinal tract (lamina propria) and develop from signals that are derived from the microbiota. Further investigations studying microbiota composition changes and concomitant variation in Th17 abundance in articular and mucosal compartment during colitis and/or arthritis could pave the way for new therapeutic strategies in EIM related IBD treatment through manipulation of the gut microbiota (probiotics, prebiotics for example).

## Supporting information

S1 Table Primers used to measure gene expression in colon and forepaws of mice. (DOCX)

S1 Text Supplementary materials and methods. (DOCX)

S1 Fig Evaluation of gut inflammation in mice (N = 8). Follow-up of colitis score (A), body-weight gain (B), fecal lipocalin-2 by ELISA (C) and colons length on day 41 (D) Histological findings (E) and scoring (F) in the four studied groups. Arthritis was induced by intradermal injection of CII at D0 and a boost was performed at D21 by intra-peritoneal injection of CII. Arthritis was induced by injection of CII (200μg) at D0 at the basis of the tail and a boost of 100μg CII was performed at D21 by intra-peritoneal injection. Colitis was induced by oral intake of 3% DSS in drinking water from D14 to D 21. Data are expressed as mean ± SEM, # P<0.05 colitis group *versus* control group. * P<0.05 arthritis + colitis group *versus* colitis group.
(TIFF)

S2 Fig **Assessment of intestinal inflammatory by measurement of KC (CXCL1) (A) and TNFα (B)** expression at day 41 in the colon of control, arthritic, colitic or arthritic and colitic mice (N = 5 per group). Arthritis was induced by intradermal injection of CII at D0 and a boost was performed at D21 by intra-peritoneal injection of CII. Arthritis was induced by injection of CII (200μg) at D0 at the basis of the tail and a boost of 100μg CII was performed at D21 by intraperitoneal injection. Colitis was induced by oral intake of 3% DSS in drinking water from D14 to D 21. Expression of cytokines was analyzed by real time PCR. Data are expressed as mean ± SEM.
(TIFF)

S3 Fig Composition of fecal microbiota in this model. (A) Differential analysis between arthritis and colitis + arthritis groups at day 30. (B) Differential analysis between arthritis and colitis + arthritis groups at day 41.
(TIFF)
