# Supplementary material for: Experimental colitis delays and reduces the severity of collagen-induced arthritis in mice
Source: PLoS One. 2017 Sep 19;12(9):e0184624. doi: 10.1371/journal.pone.0184624 (PMC5604972; doi:10.1371/journal.pone.0184624)
Supplement: S1 Table — (DOCX) [file pone.0184624.s001.docx]

| Gene | Forward | Reverse |
| --- | --- | --- |
| S29 | GGA-GTC-ACC-CAC-GGA-AGT-T | GCC-TAT-GTC-CTT-CGC-GTA-CT |
| IL-1β | TGT-GAA-ATG-CCA-CCT-TTT-GA | GTG-CTC-ATG-TCC-TCA-TCC-TG |
| iNOS | CCT-TGG-TGA-AGG-GAC-TGA-GC | CAA-CGT-TCT-CCG-TTC-TCT-TGC |
| KC (CXCL1) | AGA-CCA-TGG-CTG-GGA-TTC-AC | AGT-GTG-GCT-ATG-ACT-TCG-GT |
| TNFα | GAT-CGG-TCC-CCA-AAG-GGA-TG | TGG-TTT-GTG-AGT-GTG-AGG-GTC |
