# Supplementary material for: Experimental colitis delays and reduces the severity of collagen-induced arthritis in mice
Source: PLoS One. 2017 Sep 19;12(9):e0184624. doi: 10.1371/journal.pone.0184624 (PMC5604972; doi:10.1371/journal.pone.0184624)
Supplement: S1 Text — (DOCX) [file pone.0184624.s002.docx]

## Experimental colitis

### Clinical assessment of colitis

Daily, the severity of colitis was evaluated by the disease activity index (DAI). The DAI was calculated with the body weight loss (0 = no weight loss, 1 = 0%-5% weight loss, 2 = 5%-10% weight loss, 3 = 10%-20% weight loss, 4 = more than 20% weight loss), feces consistency (0 = normal, 2 = loose stools, 4 = diarrhea) and the presence of blood in the feces (0 = negative, 2 = positive, 4 = important presence of blood in the feces).

### Histological assessment of intestinal inflammatory

Intestinal inflammatory was evaluated in colon. The length of colons was measured and the colons were dissected, opened longitudinally, flushed with PBS and fixed in 4% formalin for 24h and embedded in paraffin blocks. 5 µm thick sections were stained with hematoxylin, eosin. Histological examination was carried out blindly and the severity of colitis was assessed according to the presence of acute/chronic inflammatory, cryptic abscesses and ulceration and of the architecture, each parameter was scored from 0 (normal) to 3 (important presence).

## Detection of Lipocalin-2 in feces by Enzyme-Linked Immunosorbent Assay (ELISA)

Feces were collected at days 8, 21, 30 and 41 of development of arthritis and frozen at -80°C until use. Each feces was reconstituted in 1ml of PBS containing 0,1% Tween 20 and grinded twice at 6000rpm for 30s with Precellys Lysing Kit. Samples were centrifuged for 10 minutes at 12,000rpm and the supernatants containing the proteins were collected and stocked at -20°C until analyze. Lipocalin-2 was measured using ELISA kit based on the manufacturer’s instructions (R&D systems). Samples removed at day 8 and all samples of groups “control” and “arthritis” were diluted to 50-fold, and samples of groups “colitis” and “colitis and arthritis” at days 21, 30 and 41 were diluted to 10,000-fold.

## Measurement of mediator expression in colons by reverse transcription polymerase chain reaction (RT-PCR)

At sacrifice, colons of mice were preserved in trizol and were frozen at -80°C until use. Colons were grinded with Precellys Lysing Kit in 1ml of trizol at 6500rpm for 30s. Samples were centrifuged at 12,000g for 10min at 4°C and 200µl of chloroform by milliliter of trizol were added to the supernatant. Samples were centrifuged at 12,000g for 15min at 4°C, 500µl by milliliter of trizol of isopropanol were added to the aqueous phase and they were incubated over night at -20°C. RNAs were pelleted by centrifugation at 12,000g for 10min and they washed with 70% ethanol twice. Finally, RNAs were taken up in water.

Reverse transcription was realized with 2µg of RNAs, 4µl of RT buffer, 2µl of dNTP at 5mM, 2µl of DTT at 100mM, 1µl of hexa primers at 0,2µg/µl and 1µl of M-MLV at 200u/µl (Invitrogen). Samples were incubated at 37°C for 1h30 and at 95°C for 5min.

For PCR, samples were diluted to one-tenth for S29 and to fifth for genes of interest (KC, TNFα). PCR was realized with 5µl of samples, 10µl of SYBR, 1µl of each primer at 10µM and 3µl of water.
