# Supplementary figures and images for: Experimental colitis delays and reduces the severity of collagen-induced arthritis in mice
Source: PLoS One. 2017 Sep 19;12(9):e0184624. doi: 10.1371/journal.pone.0184624 (PMC5604972; doi:10.1371/journal.pone.0184624)

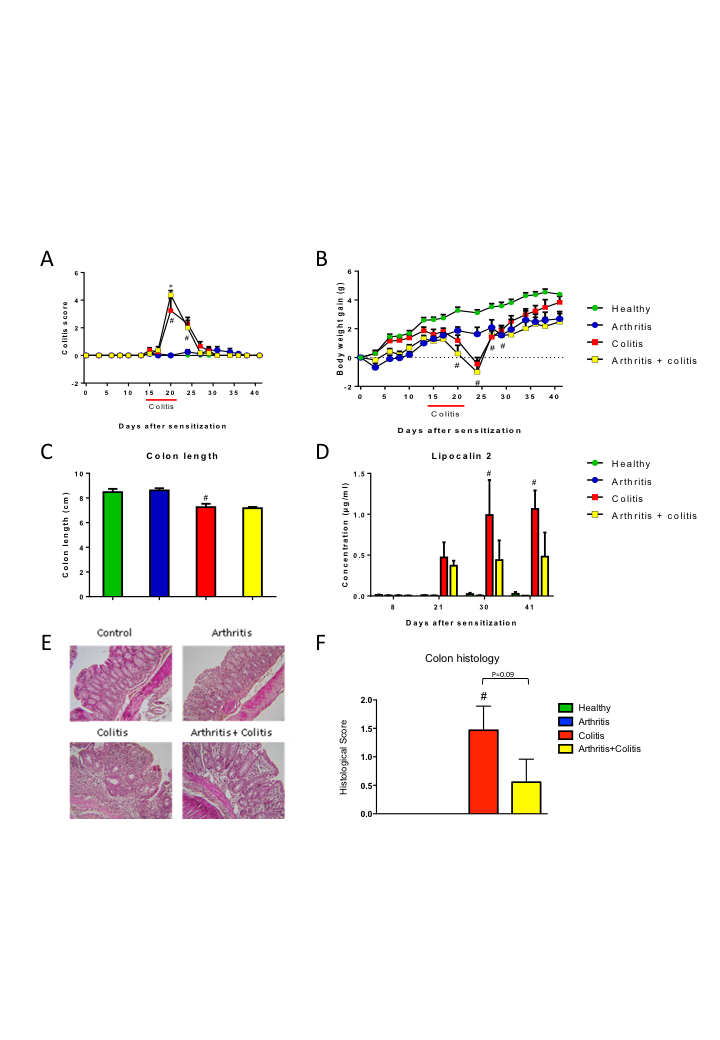

Supplement: S1 Fig — Follow-up of colitis score (A), body-weight gain (B), fecal lipocalin-2 by ELISA (C) and colons length on day 41 (D) Histological findings (E) and scoring (F) in the four studied groups. Arthritis was induced by intradermal injection of CII at D0 and a boost was performed at D21 by intra-peritoneal injection of CII. Arthritis was induced by injection of CII (200μg) at D0 at the basis of the tail and a boost of 100μg CII was performed at D21 by intra-peritoneal injection. Colitis was induced by oral intake of 3% DSS in drinking water from D14 to D 21. Data are expressed as mean ± SEM, # P<0.05 colitis group versus control group. * P<0.05 arthritis + colitis group versus colitis group. (TIFF) [file pone.0184624.s003.tiff]

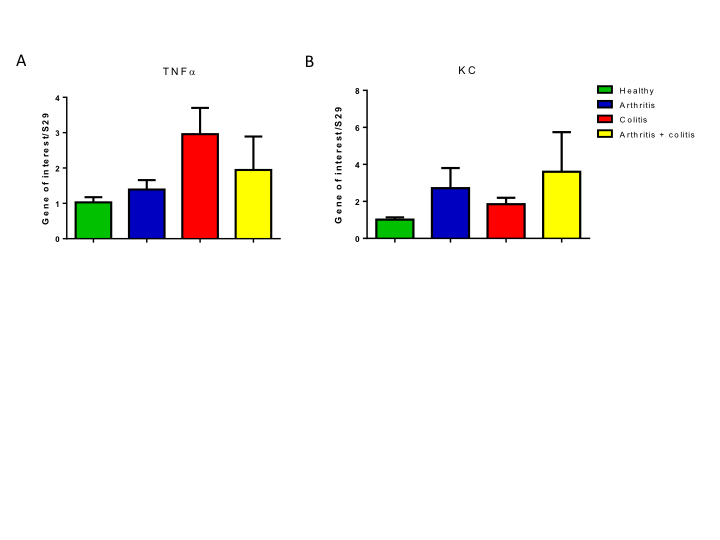

Supplement: S2 Fig — Assessment of intestinal inflammatory by measurement of KC (CXCL1) (A) and TNFα (B) expression at day 41 in the colon of control, arthritic, colitic or arthritic and colitic mice (N = 5 per group). Arthritis was induced by intradermal injection of CII at D0 and a boost was performed at D21 by intra-peritoneal injection of CII. Arthritis was induced by injection of CII (200μg) at D0 at the basis of the tail and a boost of 100μg CII was performed at D21 by intraperitoneal injection. Colitis was induced by oral intake of 3% DSS in drinking water from D14 to D 21. Expression of cytokines was analyzed by real time PCR. Data are expressed as mean ± SEM. (TIFF) [file pone.0184624.s004.tiff]

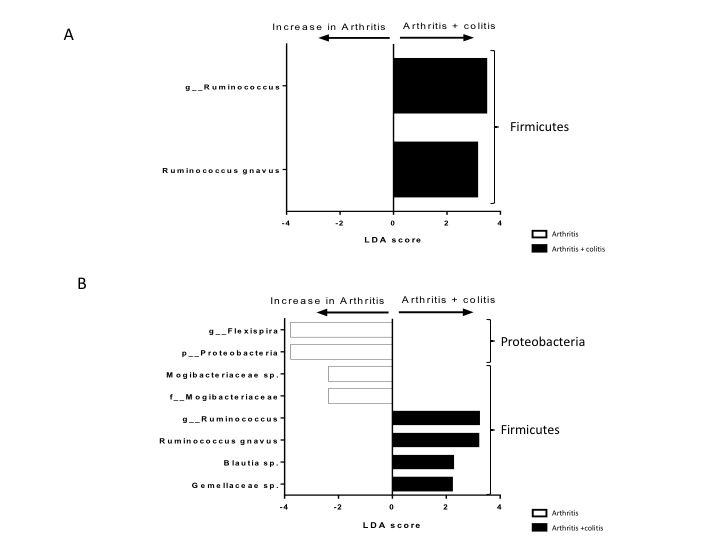

Supplement: S3 Fig — (A) Differential analysis between arthritis and colitis + arthritis groups at day 30. (B) Differential analysis between arthritis and colitis + arthritis groups at day 41. (TIFF) [file pone.0184624.s005.tiff]
